# A novel surgical management for male infertility secondary to midline prostatic cyst

**DOI:** 10.1186/s12894-015-0015-8

**Published:** 2015-03-14

**Authors:** Gong Cheng, Bianjiang Liu, Zhen Song, Aiming Xu, Ninghong Song, Zengjun Wang

**Affiliations:** State Key Laboratory of Reproductive Medicine and Department of Urology, The First Affiliated Hospital of Nanjing Medical University, Nanjing, 210029 China

**Keywords:** Male infertility, Midline prostatic cyst, Transurethral resectoscopy, Seminal vesiculoscopy

## Abstract

**Background:**

To summary the procedure and experience of a novel surgical management for male infertility secondary to midline prostatic cyst (MPC).

**Methods:**

From February 2012 to February 2014, 12 patients were diagnosed with PMC by semen analysis, seminal plasma biochemical analysis, transrectal ultrasonography (TRUS), and pelvic magnetic resonance imaging (MRI). All patients underwent the transurethral unroofing of MPC using resectoscope, the dilation of ejaculatory duct, and the irrigation of seminal vesicle using seminal vesiculoscope. All patients were followed up at least 3 months after operation.

**Results:**

Preoperative semen analyses of 12 patients showed oligoasthenozoospermia (5/12) or azoospermia (7/12), low semen volume (0–1.9 mL), and low pH level (5.5-7.0). Preoperative seminal plasma biochemical analyses showed reduced semen fructose. TURS and MRI revealed a cyst lesion located in the midline of prostatic. After 3 months follow up, the semen quality of 80% patients (4/5) with oligoasthenozoospermia improved obviously. The spermatozoa were present in the semen in 5 of 7 cases with azoospermia. In one patient, the spermatozoa occurred in the urine after ejaculation.

**Conclusions:**

Surgical management using transurethral resectoscopy and seminal vesiculoscopy is effective, minimally invasive, and safe for male infertility secondary to MPC.

## Background

Male infertility affects about 8% of couples around the world [1]. Midline prostatic cyst (MPC), a rare but surgically correctable disease, is deemed to cause ejaculatory duct obstruction (EDO) which accounts for approximately 1-5% of male infertility [2]. Most MPCs are nonsymptomatic. Semen analysis, transrectal ultrasonography (TRUS) and pelvic magnetic resonance imaging (MRI) can help to diagnose MPC. For treatment, cyst puncture and simple endoscopic section are widely used. However, the efficacy is often poor. The present study shows a novel surgical management for male infertility secondary to MPC.

## Methods

Approval for this study was granted by the ethics committee of Nanjing Medical University (China) and informed written consent was received from all participants.

### Patients

**Figure 1:**
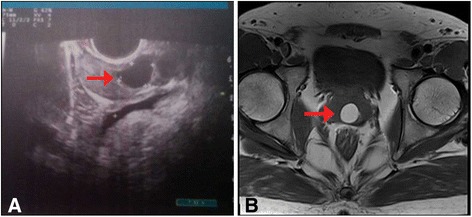
**The representative images of MPC. A**, TRUS; **B**, pelvic MRI. The arrow indicates the cystic lesion.

From February 2012 to December 2013, 12 patients were recruited at Department of Urology, The First Affiliated Hospital of Nanjing Medical University. The patients were aged 18–40 years. All of them complained of infertility 2–10 years after marriage. Two cases had hematospermia. Preoperative semen analyses of 12 patients showed oligoasthenozoospermia (5/12) or azoospermia (7/12), low semen volume (0–1.9 mL), and low pH level (5.5-7.0). Preoperative seminal plasma biochemical analyses showed reduced semen fructose. TURS (Figure 1A) and MRI (Figure 1B) revealed a cyst lesion located in the midline prostate.

### Procedures

**Figure 2:**
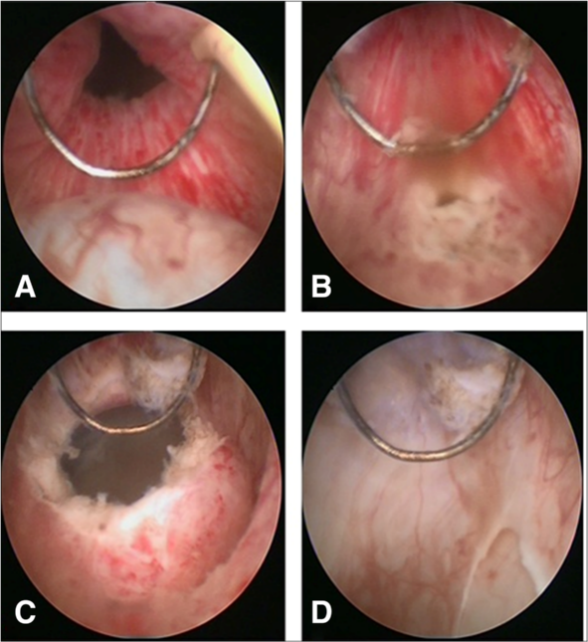
**Transurethral unroofing of MPC. A**, the cyst is visualized through the resectoscope. **B** and **C**, the ridgy posterior wall of the urethra is resected for unroofing the MPC. **D**, TURED is performed to make the ejaculatory duct unobstructed.

**Figure 3:**
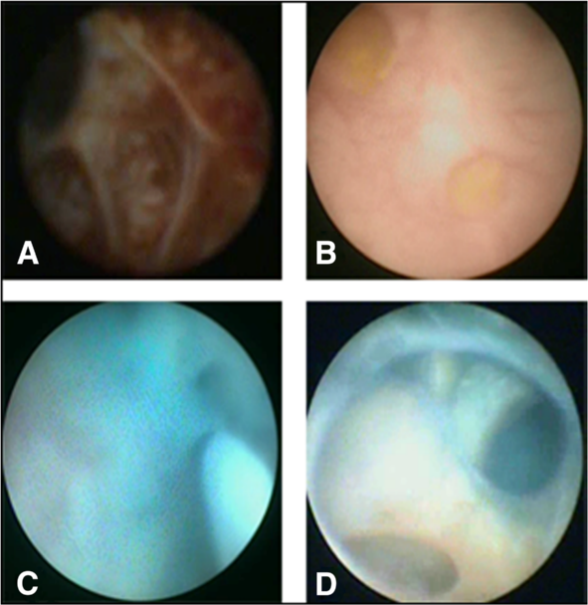
**Transurethral irrigation of seminal vesicle. A**, seminal vesiculitis contains the congestive wall, and milky, yellow or pink vesicle fluid filled with flocculent turbidity and dark blood clots. **B**, seminal vesicle stones. **C** and **D**, the seminal vesicle is washed clearly and irrigated using a levofloxacin solution.

The patients were placed under general anesthesia in the dorsal lithotomy position. Transurethral unroofing of MPC was performed using the F26 resectoscope. First, the resectoscope was inserted into prostatic urethra for preliminary visualization of the cyst (Figure 2A). Then the ridgy posterior wall of the urethra was resected for unroofing the MPC (Figure 2B and C). If the ejaculatory duct opening was not obvious, transurethral resection of the ejaculatory duct (TURED) was performed to make the ejaculatory duct unobstructed (Figure 2D). Lastly, the dilation of ejaculatory duct and the irrigation of seminal vesicle were performed using F7 seminal vesiculoscope according to our previous report [3]. Under the guidance of a zebra guidewire, the endoscope was inserted into the ejaculatory ducts and seminal vesicles at the help of hand-controlled intermittent water perfusion dilation. The seminal vesicles usually contained the congestive wall, and milky, yellow or pink vesicle fluid filled with flocculent turbidity and dark blood clots (Figure 3A). In some cases, seminal vesicle stones were even found (Figure 3B). The seminal vesicles were irrigated using a levofloxacin solution (Figure 3C and D). After operation, a urethral Foley catheter was remained overnight. All patients were required to refrain from ejaculation at least two weeks and followed up at least 3 months.

### Outcomes analysis

Preoperative and postoperative serum sex hormone levels were recorded. Preoperative and postoperative semen qualities were monitored using a computer-assisted semen analyzer (IVOS; Hamilton-Thorne, Beverly, MA, U.S.).

## Results

All operations were completed without conversion to open surgery. There were no severe complications such as rectal injury and urethral sphincter damage. The average length of hospitalization was 3 days. Serum sex hormone levels of all patients prior to and after surgery were within the normal range. The preoperative and postoperative semen parameters were shown in Table 1. After 3 months follow up, the semen quality of 80% patients (4/5) with oligoasthenozoospermia improved obviously. The spermatozoa were present in the semen in 5 of 7 cases with azoospermia. In one patient, the spermatozoa occurred in the urine after ejaculation.

**Table 1:** **The preoperative and postoperative semen parameters of 12 cases**

	**Preoperative**		**Postoperative**	
	**Concentration (10** ^**6**^ **/mL)**	**Grade a + b (%)**	**Concentration (10** ^**6**^ **/mL)**	**Grade a + b (%)**
Oligoasthenozoospermia 1	11	33.3	45	60.0
Oligoasthenozoospermia 2	6	12.7	21	48.5
Oligoasthenozoospermia 3	15	26.1	73	66.5
Oligoasthenozoospermia 4	2	12.3	4	11.1
Oligoasthenozoospermia 5	9	22.4	50	59.6
Azoospermia 1	0	0	21	62.3
Azoospermia 2	0	0	17	52.3
Azoospermia 3	0	0	0	0
Azoospermia 4	0	0	32	68.7
Azoospermia 5	0	0	10	45.5
Azoospermia 6^*^	0	0	0	0
Azoospermia 7	0	0	43	57.9

^*^The spermatozoa occurred in the urine after ejaculation.

## Discussion

MPC is previously thought to be Mullerian duct cyst, causing EDO and male infertility through oppressing ejaculatory duct [4]. Recent researches reveal that not all cystic lesions are located in the midline prostate originate from Mullerian duct remnant. Some cases are just the cystadenoma or the simple cyst of prostate [5]. Such cyst in the midline prostate should be termed as prostatic utricular cyst or cystic dilation of prostatic utricle, depending on whether an outlet to the urethra exists.

Despite the embryologic or histological origin, MPC is a surgically correctable disease. Many MPCs are asymptomatic, or have some non-specific symptoms such as perineal pain, hematospermia, and painful ejaculation. One of the most important and serious outcomes caused by cystic lesion oppression is EDO and male infertility. Semen analysis of these patients often showed low semen volume and pH level, reduced semen fructose, oligoasthenozoospermia, and even azoospermia.

Vasoseminal vesiculography is the golden standard for diagnosis. Percutaneous testicular sperm aspiration (PTSA) is helpful to identify the type of MPC. However, these methods are invasive and complicated. TURS is preferred for simple and noninvasive characteristics. It can offer greater details about the relationship of prostatic, seminal vesicle, and ejaculatory duct [6]. Pelvic MRI not only clearly shows the anatomy of prostatic, seminal vesicle, and ejaculatory duct, but also helps to judge the nature of cyst fluid [7].

The treatment for MPC is still controversial. Some researchers claimed that treatment should only be performed on symptomatic or infertile patients since almost 60% of cases with MPC did not experience any cyst-related symptoms or fertility impairment [8]. Invasive procedures include transperineal or transrectal puncture and endoscopic section of the utricle meatu [9]. However, puncture therapy has a high recurrence rate while endoscopic incision faces persistent post-operative severe oligozoospermia or azoospermia [10]. We speculate that MPC may cause to seminal vesiculitis and further promote the abnormal semen quality through oppressing ejaculatory duct and causing semen stasis. Therefore, the dilation of ejaculatory duct and the irrigation of seminal vesicle using seminal vesiculoscope were performed after transurethral unroofing of the cyst in present study. A previous study achieved a better result for MPC with male infertility using transurethral endoscopic incision, in which a pregnancy rate was 30.8% (8/26) [10]. However, other reports showed a poor efficacy of unroofing of the cyst [11]. The present study is delightful with a pregnancy rate of 41.7% (5/12) and an obviously improved semen quality of 75% (9/12). One patient with azoospermia had spermatozoa in the urine after ejaculation. For the two patients of poor efficacy, seminal vesicle impairment due to long term semen stasis might cause inadequate transfer of semen quality [12]. In conclusion, our data showed that the novel surgical management is effective, minimally invasive, and safe for male infertility secondary to MPC.

The followings are our endoscopic experiences. Sometimes, it is difficult to find the opening since the ejaculatory duct is oppressed by MPC or covered by inflammatory tissues [3,13]. Increasing the velocity of water perfusion and using a zebra guidewire as the guidance are helpful to find the openings. Otherwise, vas deferens puncture and injection of methylene blue, combined with the transurethral endoscope, are helpful to observe the ejaculatory duct openings. After observing the seminal vesicle lumen carefully, appropriate therapies can be implemented. Inflammatory semen can be treated with antibiotics irrigation into seminal vesicle. Stones can be removed through holmium laser lithotripsy and forceps.

## Conclusions

Surgical management using transurethral resectoscopy and seminal vesiculoscopy has good clinical outcomes for improving the semen quality. It is effective, minimally invasive, and safe for male infertility secondary to MPC.

## Abbreviations

MPC: Midline prostatic cyst
EDO: Ejaculatory duct obstruction
TRUS: Transrectal ultrasonography
MRI: Magnetic resonance imaging
TURED: Transurethral resection of the ejaculatory duct
